# Strictures, in Reply to Mr. Wright's Objections to the Operation of Puncturing the Membrana Tympani

**Published:** 1819-04

**Authors:** J. Vale Asbury

**Affiliations:** Member of the Royal College of Surgeons, and Licentiate of the Society of Apothecaries in London.


THE LONDON

### Medical and Physical Journal.

4 OF VOL. XLI.] APRIL, 1819. [NO. 242.

"For many fortunate discoveries in medicine, and for the detection of numerous errors, the world is indebted to the rapid circulation of Monthly Journals; and there never existed any work to which the Faculty in EUROPE and AMERICA were under deeper obligations than to the Medical and Physical Journal of London, now forming a long, but an invaluable, series."—RUSH.

*For the London Medical and Physical Journal.*

### *Strictures, in Reply to Mr. Wright's Objections to the Operation of puncturing the Membrana Tympani;* by J. VALE ASBURY, Esq. Member of the Royal College of Surgeons, and Licentiate of the Society of Apothecaries in London.

I HAVE recently noticed, in "an Essay on the Human Ear," by Mr. Wright of Bristol, objections to the operation of puncturing the membrana tympani in those cases of deafness which arise from obstruction of the eustachian tubes; and a dissent from the conclusion drawn in my observations on some particular states of the organ of hearing, which were published in the Medical Repository for October 1817, in those cases where the faculty of hearing continues after the membrana tympani is destroyed, and the ossicula auditus have come away. In these particular instances, I suppose that the membrana fenestræ ovalis must remain entire; otherwise, the aqueous contents of the labyrinth would escape, whereby all power of hearing is completely destroyed. In reply to which, this gentleman argues in a manner somewhat singular:—"Now, when, from scarlatina or cynanche tonsillaris, the bones come away with the membrana tympani, it arises from the ulceration that affects the whole membranous lining of the mouth and pharynx; and this lining being continued, and extending itself down the eustachian tube into the cavity of the tympanum, the diseased action must be general; and, the stapes being fixed by fine membranous filaments into the cavity of the fenestra ovalis, which are destroyed by the ulceration, it is the first bone, generally, that comes away: therefore, the covering which, it has been observed, the base of this bone with its membrane affords to the fenestra ovalis, is necessarily destroyed; and no vibration, it will seem, can therefore take place by that aperture, for want of the necessary membrane."—p. 76. Unless Mr. Wright, at the same time, had proved that, when the membrana fenestræ ovalis is destroyed by ulceration, the power of hearing continues in a greater or less degree, his argument, it will be found, does not militate against the principle which I advanced; viz.—when the hearing continues after the bones of the tympanum and its membrane are destroyed, the membrana fenestræ ovalis must remain entire; though he venture to assert, in the same paragraph, "but I cannot agree in the conclusion this gentleman makes upon his own reasoning." On the contrary, this writer coincides with the opinion of Mr. Cruikshanks, and observes, in p. 77 of the above essay, —"His [Mr. Cruikshank's] opinion was clearly the same as mine—that the destruction of the fenestra ovalis would obliterate hearing;" and the experiments, moreover, which led to this opinion "were made on dogs, who were found to have some sense of hearing after the membrana tympani, malleus, and incus, were destroyed."—p. *ubi sup.* Here it is quite obvious that Mr. Wright, when commenting on my remarks, makes, in his view of the subject, a distinction without a difference; for, if he admit, on the one hand, "that the destruction of the membrana fenestræ ovalis would obliterate hearing," he cannot consistently dissent, on the other, from the conclusion that, "in these cases [i. e. where the hearing continues,] the membrana fenestræ ovalis must remain entire, otherwise the aqueous contents of the labyrinth would escape, whereby all power of hearing is completely destroyed."*

From this writer's remark, that "no vibration, it will seem, can take place by that aperture [fenestra ovalis], for want of its necessary membrane," and also from his omission (when describing the structure of the internal part of the organ of hearing) of this anatomical fact, that the labyrinth contains a fluid: from these two circumstances it would appear, that he conceives a vibration in the membrana fenestræ ovalis, or membrana fenestræ rotundæ, as the essential medium for conveying the impressions of sound to the auditory nerve; but a minute investigation of the mode in which this nerve is distributed, and of the membranes and fluid of the labyrinth which are immediately in contact with and support this distribution, will prove that something

---

* From the paper I published, *on the Function and some particular States of the Organ of Hearing; with a description of a newly-invented Instrument for puncturing the Membrana Tympani*, in the London Medical Repository, vol. viii. p. 199.

more is required to convey the impulse of sound to the nervus auditorius than a mere vibration of the membranes seated in the fenestra ovalis and the fenestra rotunda. I will suppose, for a moment, the labyrinth to consist simply of a bony box complete on all sides except the external, in which the deficiency of the bony case is supplied by two distinct membranes occupying two separate apertures, and which are technically termed the fenestra ovalis and fenestra rotunda. This box is completely filled with a fluid and membranous canals, which, on entering it by the fenestra ovalis, take a very intricate course, (hence this part of the organ is denominated the labyrinth,) and ultimately terminate at the fenestra rotunda. On these membranous canals, and on two sacculi, which have a particular situation in the labyrinth, there will be found the ultimate distribution of the auditory nerve. The aqueous fluid of the labyrinth suspends and maintains the membranous canals and sacculi, which are connected to the surrounding bony case by a loose cellular tissue, in a fit state for receiving the expansions of the auditory nerve on them; thus enabling the impulsions of sound to make an impression on these expansions; and this fluid is confined in the labyrinth by the membrana fenestræ ovalis and the membrana fenestræ rotundæ, which complete the external side of the box. In this view of the interior of the organ, the undulatory rays of sound, striking against the membrana fenestræ ovalis, communicate an undulatory motion to the aqueous fluid of the labyrinth, which undulation is received by the expanded extremities of the nerve, and thence transmitted through the branches of this nerve to the brain. These undulations in the fluid of the labyrinth are rendered perfect and uniform by the membrane that occupies the fenestra rotunda, which, from its elasticity, yields in some degree at the time the impulse of sound is given on the membrane of the fenestra ovalis; without this particular construction, the vibrations of sound are in themselves too feeble to make any impression on the incompressible fluid within the labyrinth; and, supposing a compressible substance in this part of the organ, it would then be unfit for passing the impulsions of sound forward to the expansions of the nerve. It is now evident that the great use of the two membranes in the external side of the box is to confine the aqueous fluid in the labyrinth, which fluid is the medium of communication between the auditory nerve and the fenestra ovalis; and, when either of these membranes are destroyed, the box itself becomes incomplete, the fluid in it escapes, the membranous canals contract, the expansions of the nerve are lost, and thus the individual becomes permanently deaf. From these considerations we can readily conclude "how far the membrana fenestræ rotundæ may (when the membrana fenestræ ovalis is destroyed) convey the sounds partially," which, it is the opinion of Mr. Wright, "we are yet to learn."

The objections which this gentleman makes to puncturing the membrana tympani, in those cases of deafness arising from an obstruction of the eustachian tubes, are, it is obvious, more founded on theory than on practice; they are as follow:—

"It is well known that no atmospheric air can naturally pass into the cavity of the tympanum, except through the eustachian tube; by which, I presume, it becomes regulated in temperature to those parts to which it is eventually destined.

From the anatomy of the ear, it appears that the two fenestræ, or apertures, leading to the more sensitive parts of the organ, are each protected by a membrane; and, both by the situation of that, as well as the apertures themselves being opposite to the membrana tympani, I am led to conclude they are not calculated to receive immediate, but reflect, sounds."

This last objection, which is founded on "the anatomy of the ear," and allowing the above statement of it to be accurate, would rather make for than against the operation; for, if the two membranes on the external side of the labyrinth were directly opposite to the membrana tympani, the natural conclusion is, that both of them are certainly destined to receive immediate impressions from without, and not to reflect them from within the organ, where the impulsion ought to pass onward to the auditory nerve. Again, admitting that a power of reflection does take place from the membrana fenestræ ovalis and the membrana fenestræ rotundæ, these membranes, if they be situated opposite to the inlet of sound, must, in the first instance, receive immediate impressions. But a more correct view of the membranes in the fenestra ovalis and fenestra rotunda will show that the membrana fenestræ ovalis, *only*, is immediately opposite to the membrana tympani; while the membrana fenestræ rotundæ faces the mastoid cells, making an obtuse angle with the plane of the membrana tympani: so it would appear that the latter membrane is calculated to reflect, and the former to receive, the sonorous rays. As the above objections, however, as well as that "founded on the chemical properties and changes atmospheric air undergoes by passing into the human body," have their origin merely in supposition, it is only necessary for me to observe further, that practice, in several cases, has proved that the membrana tympani may be punctured, when the operation is judiciously performed, without the slightest bad consequences arising to the internal parts of the organ, either directly from the operation itself or from the temperature of the air, or from the direct action of the sonorous vibrations on the delicate membranes of the labyrinth. And Mr. Wright himself observes, that "many persons, beside the North-American Indians, can drive tobacco-smoke from the mouth through the meatus externus, and yet have acute hearing;" which, being viewed impartially, is of itself sufficient to put aside all his supposed objections to the operation. For, if it be established as a fact, (and, as cases are on record in proof of it, I do not see on what ground it can be denied,) that the sense of hearing continues when there is an opening in the membrana tympani, it is of very little consequence how this opening came there; and, however sceptical Mr. Wright is, or may remain, "as to its being more than a *lusus naturæ*," we are perfectly justified in making an artificial aperture, where deafness arises from a permanent obstruction of the eustachian tubes, so as to restore individuals to a greater perfection of hearing than they can, in any other way, experience when labouring under such obstructions.

My own practice, and that of others, warrants this conclusion. I have punctured the membrana tympani in two cases, and I succeeded instantly in restoring the faculty of hearing to the utmost of my expectations; though the sense of hearing, after the operation had been performed, was not quite so perfect as it is in a natural state of the organ, yet the degree of benefit derived from it was such as fully to authorize a repetition of the practice in any subsequent case of a similar nature; and the remaining imperfection of this faculty was not a hindrance to the enjoyment of a free conversation. But this gentleman observes, that, "by perforating the membrana tympani, a painful sensibility of hearing takes place, which goes off by degrees, till the faculty is nearly or quite obliterated." I trust I do not misapprehend the writer in supposing that he means—till the faculty *of hearing* is nearly or quite obliterated; and, if he do not mean the faculty of hearing, his own arguments will again prove, independent of those I could adduce, and others that may be gleaned in the above observations, that an opening may exist in the membrana tympani without any material diminution in the auditory sense. True it is that a painful sensibility of hearing takes place immediately on perforating the membrane, which, as the parts internally become accustomed to the new impressions of sound, gradually goes off, leaving the patient's hearing greatly amended; and this amendment continues so long as the artificial opening remains pervious. But this increase of sensibility does by no means form any objection to the performance of the operation; because it has been, and may be obviated, by introducing a piece of cotton-wool into the external meatus, so as to prevent for a time the rapid access of air into the tympanum; and, by taking this precaution, no injury to the interior parts of the organ, or even inconvenience to the patient, can arise.

It is a singular fact that Mr. Wright should, in his objections to a practice that in some cases has been successful, but which he himself never found to succeed, judiciously omit to point out, in his enumeration of causes occasioning obstruction of the eustachian tubes, that cause which would in itself produce an obliteration of these passages; and in which case the operation, if carefully performed, will never fail to restore, in a great degree, the original power of hearing:—I mean inflammation of the eustachian tubes, terminating in a secretion of coagulable lymph, which, when coagulated, completely closes them; and then the blood-vessels and nerves of the membrane lining these tubes shoot through this coagulated lymph, forming a new fleshy substance that entirely obliterates the passages, precisely in the same manner, and as permanently, as a deep wound, made in any fleshy part of the body by a sharp instrument, is healed by the first intention. In this instance, Mr. J. Wathen's plan of injecting the eustachian tubes, and which Mr. Wright advocates, will never succeed in restoring the passages, any more than the act of injecting the cicatrix of a wound, after it is healed, will again produce a wound. What, then, remains to be done in these cases? The cavity of the tympanum, with an obstruction of the eustachian tubes, is similarly circumstanced to a common drum with a cork placed in the aperture on the side of it, and hermetically sealed, which, on beating it, will be found greatly diminished in tone; but, by removing the cork, and allowing a free communication between the air without and that which is within the drum, its full tone is again restored. So it is with the tympanum, or drum of the ear: an obliteration of the eustachian tubes cuts off the communication between the external air and that contained in the cavity of the tympanum; and, being thus confined, its undulation is prevented, so that the sonorous impulsions cannot wholly be transferred to the fluid of the labyrinth, and, in consequence, the sense of hearing is greatly defective: but, if this ear-drum be made to communicate with the atmospheric air by a small aperture in its membrane, a due impulsion of sound is given to the aqueous fluid in the labyrinth, the expanded nerve receives it, and the individual recovers the sense of hearing.

The writer of the above essay, however, does not conceive "the operation is ever serviceable;" and, as a concluding paragraph in support of his opinion, he observes—

"Where obliteration or closure of the eustachian tubes has taken place, it arises chiefly from the causes I have mentioned, except when a nasal polipus may, by protruding into the pharynx, occasion an obstruction: the latter, of course, will be relieved by the removal of the tumor; and, from the former causes, the ulceration is so extensive that it mostly produces the effects I have just mentioned, and, in such cases, little, if any thing, can be attained by the operation."

With all due deference to Mr. Wright, it is necessary, in order to take an impartial view of the case in point, to analyze this passage. The causes to which this gentleman alludes are "from the effects of cold, and a variety of undeterminate causes;" and, in the first extract which I have given at the commencement of these observations, it will be seen that he also has in consideration the ulceration that affects the whole membranous lining of the mouth and pharynx: it is from the latter cause that he observes, "the ulceration is so extensive that it mostly produces the effects I have just mentioned;" and these effects are (it will be found on examining the above extract) ulceration extending from the throat along the eustachian tube and over the cavity of the tympanum, thus destroying its membranes, and also the escape of the bones from the tympanum:—"in these cases (he continues) little, if any thing, can be attained by the operation." Who would propose any measure to puncture that which does not exist?

The difficulty this writer found in explaining away the benefit of the operation in a practical case, which I have published, is manifest, when we consider how much he has deviated, in his mode of arguing, from the established laws of inflammation. Ulceration of the eustachian tube, either from scarlatina or cynanche tonsillaris, is indeed an exceedingly unfrequent occurrence, as may be inferred from the anatomical formation of these parts; for, when inflammation arises in the membrane lining this tube, a thickening of it is produced, and, from the original narrowness of the passage, a slight secretion of coagulable lymph closes it by adhesion; the effect of which is to arrest the progress of the inflammation in the tube. By the same rule, we continually find, on opening the chest *post mortem*, adhesion between the pleura lining the chest and that covering the lungs; which is the result of inflammation occurring in contiguous surfaces. And from the same inflammatory action, producing an obliteration of the eustachian tubes, arose that case of deafness, after a violent attack of scarlatina, in which I punctured the membrana tympani with success: but they who are adverse to the operation wave the inflammatory process altogether, (which is the only cause, as far as our knowledge yet extends, that produces the species of deafness which can only be benefitted by an artificial opening in the membrana tympani,) and proceed to argue on an extremely rare process of ulceration, which, when it does occur, must completely destroy the organ of hearing; and then they arrive at the conclusion, that, in such cases, "little, if any thing, can be attained by the operation." Under these circumstances, we may readily infer in what manner "the practice is falling into considerable disrepute."

Fortunately, however, it is not sufficient to prove, because the operation has been unsuccessful in some cases, or that it should fail to restore the hearing under the management of one practitioner, "that the practice is altogether bad;" for it is possible, by a candid exposition of facts, to exhibit this mode of practice in a much more favourable light than some are wont to do. Lord Bacon observes, in "The Advancement of Learning," book ii.—"I take it, those things are to be held possible which may be done by some one, though not by every one; and which may be done by many, though not by any one."

*Enfield; January* 1819.

---

*For the London Medical and Physical Journal.*

